# Determinants of willingness to pay for community-based health insurance scheme among households in rural community of southern Ethiopia

**DOI:** 10.1186/s12913-023-10406-w

**Published:** 2023-12-06

**Authors:** Yonas Abebe, Fanuel Belayneh

**Affiliations:** 1 Ministry of Health, Ethiopia, Addis Ababa, Ethiopia; 2 School of Public Health, College of Medicine and Health Sciences, Hawassa University, Hawassa, Ethiopia

**Keywords:** Willingness, Community-based health insurance, Rural households, Gombora District, Ethiopia

## Abstract

**Background:**

Community-based health insurance programs are being acknowledged as effective strategies to attain universal health coverage and mitigate the financial catastrophic shock of the community. Even though Ethiopia has been focusing on the implementation and expansion of a community-based health insurance (CBHI) program since 2011, only a small number of people are enrolled, which might be attributed to a lack of willingness towards the program. The purpose of this study is to determine the willingness to pay for community-based health insurance and associated factors among households in the rural community of Gombora District, Hadiya Zone, southern Ethiopia.

**Methods:**

Using the multistage systematic random sampling technique, a sample of 421 households was chosen for a community-based cross-sectional study. The desired information was gathered using a pre-tested, structured, interviewer-administered questionnaire. The data was entered using Epi-Data V3.1 and exported to SPSS version 24.0 for statistical analysis. Bivariable and multivariable logistic regression analyses were performed to determine the variables associated with the willingness to pay for community-based health insurance.

**Results:**

The study showed that 67.1% of respondents expressed a willingness to pay for community-based health insurance. The mean amount of money they are willing to pay for the scheme is 178.41 (± 57.21) Ethiopian Birr (ETB), or 6.43 (± 2.06) USD per household per annum in 2020. Based on multiple logistic regression analysis, belonging to Rich household compared to poor (AOR: 2.78, 95% CI: 1.54, 5.03), having a household head who can read and write (AOR: 2.90, 95% CI: 1.39, 6.05), family size greater than five (AOR: 1.76, 95% CI: 1.06, 2.92), indigenous community insurance (iddir) participation (AOR: 2.83, 95% CI: 1.61, 4.96), and the presence of chronic illness (AOR: 1.94, 95% CI: 1.21, 3.12), were significantly associated with the willingness to pay for a CBHI scheme.

**Conclusion:**

Households’ willingness to pay for a CBHI scheme was found to be significantly influenced by poor household wealth status, household heads who cannot read and write, households with less than or equal to five family members, households who participate in greater or equal to two indigenous community insurance participations, and the absence of chronic illness within the household. Therefore, factors affecting households’ willingness to pay should be considered and massive community mobilization needs to be done to strengthen and increase household membership during the implementation of the CBHI scheme, especially in rural areas.

**Supplementary Information:**

The online version contains supplementary material available at 10.1186/s12913-023-10406-w.

## Introduction

### Background

Globally, financial constraints for healthcare services are increasing, which causes many individuals around the world, especially in developing countries, to suffer and die because they do not have access to even the most basic medical care. This is a result of the poor’s inability to afford medical services and their unanticipated health shock [1]. Creating a financial risk pooling mechanism that provides cross-subsidies in health systems where the ability to pay determines funding contributions and the utilization of services is based on the need for care is a crucial component of obtaining universal health coverage [2]. In many developing nations, it has been challenging to attain universal healthcare coverage (UHC), as substantial numbers of people continue to rely excessively on direct out-of-pocket (OOP) costs, such as payments for over-the-counter medications and fees for consultations and operations [3].

According to the World Health Organization (WHO), a financial catastrophe affects 150 million people annually, and more than 100 million people are pushed into poverty as a result of direct payments for services related to health care [4]. The fundamental issue in low- and middle-income countries is a lack of financial security that results in the elimination of health care and impoverishment, keeping families in a cycle of misery and ill health [5].

Community-based health insurance (CBHI) is a non-profit form of health insurance that protects the underprivileged, the unemployed, and those residing in rural areas from the high costs of seeking medical care and treatment for illness. Members of CBHI regularly pay small premiums into a collective fund [6]. It benefits from minimizing the equity gap and lowering out-of-pocket expenses, raising knowledge of the importance of insurance, fostering participant confidence through community control mechanisms, and improving use of the healthcare system [7].

Several sources provide funding for Ethiopia’s health system, including: Household out-of-pocket costs account for about 34.0% of overall health spending, which is likely to put the poor below the poverty line [8, 9].

Due to a low health care budget and a lack of advanced ways to raise money for healthcare, Ethiopia’s health care financing system is heavily dependent on high out-of-pocket expenses [10, 11].

Although the enrollment rate is low and varies between regions and pilot districts, community health insurance schemes have been developed in Ethiopia since 2011 to protect household income from illness-related expenses, which in turn increases access to and use of modern health care services [11]. Contrarily, demand surveys are rarely carried out or taken into account when constructing health insurance programs in developing nations, which has a negative impact on enrollment in many locations where CBHIs have been implemented. If CBHI is to improve access to care for the poor, it is crucial to analyze the factors of better-performing schemes, comprehend the reasons why poor households insure, and address the problems causing others to remain uninsured. The variations in membership in voluntary schemes suggest that there are factors that prevent individuals from enrolling, so this is something that needs to be addressed [12].

The annual average premium per household for the Southern Nations, Nationalities, and People’s Regional (SNNPR) of Ethiopia was 126 ETB (US$ 6.98) [13]. Before scaling up CBHI, it is important to know about the acceptance and sustainability of the scheme, but little is known about the level of willingness to pay and the factors that affect it in SNNPR in general and in Gombora district in particular. Likewise, published data on demand for CBHIS in the district and study area is lacking. On the other hand, the disparities in willingness to pay and coverage between and within regions and districts indicate that CBHI programs are more likely to be successful within particular socioeconomic contexts and circumstances [14, 15].

The health service utilization in the country remained low (34.3%) compared to most African countries [12]. According to studies, poor health care financing is a significant contributor to the country’s low health service utilization. As a result, Ethiopia has developed two policy solutions to strengthen the health-care financing system: community-based health insurance and social health insurance schemes. Community-based health insurance is a voluntary health insurance scheme that aims to improve financial access to health care services for rural communities’ informal sectors. Social health insurance, on the other hand, is a type of mandated health insurance for employees in the formal sector [14, 15]. The goals of CBHI were to promote financial access to health care services, increase resource mobilization, and improve health care quality [14, 15]. However, only a small number (12.8%) of the households were willing to pay for the program in Ethiopia [13]. Therefore, this study aimed to assess the willingness to pay for community-based health insurance and its determinants in the study area, which is in turn very important for the implementation and sustainability of the scheme in the district.

## Methods

### Study design, area and period

The investigation was conducted using a community-based cross-sectional study methodology. The study was carried out in 22 rural kebeles (“Kebele” is the lowest administrative body in Ethiopia, which comprises at least 1000 households or a population of 5000 people) in the Gombora district of the Hadiya Zone in southern Ethiopia. The district is located about 259 km south of Addis Ababa. It has a total area coverage of 48,325 ha and is geographically located between 70 33′ and 70 37′ northern latitudes and 370 35′ and 370 40′ eastern longitudes. The major economic activities of the area are mainly rain-fed subsistence mixed crop and livestock production associated with trees grown either in wood lots or in farm plots, and some kebeles use irrigation. There were 24 health posts, 1 primary hospital, and 3 operational health centers in the district. The research was carried out between January 1 and February 30, 2020.

### Study population and eligibility criteria

Gombora district was selected purposefully from the 11 districts found in Hadiya Zone in southern Ethiopia. There are 22 rural kebeles [i.e., lower administrative units in Ethiopia] in the district. The heads of all rural households in the Gombora district served as the study’s source population. The heads of households in the chosen rural kebeles in the Gombora district are chosen at random as the study population from among those source populations. All household heads who resided permanently in the study kebeles met the eligibility criteria. We excluded households that have been employed in the formal sector, since according to the health insurance proclamation of Ethiopia, such households are covered by the social health insurance scheme, which was rolled out in the middle of 2014. Based on this proclamation, Social Health Insurance (SHI) is a form of healthcare financing and management based on risk pooling to protect people from catastrophic healthcare costs. On the other hand, household heads who lived for less than six months or who were sick and unable to sustain an interview were excluded from the study.

### Sample size determination

The sample size was determined by using the single population proportion formula and considering the following assumptions: The proportion of households willing to pay for the CBHI scheme was 79.0% (*p* = 0.79), which is taken from the previous study [16], using 5% margin error at a 95% confidence interval (Z = 1.96) in order to get the sample size, which represents a true population. Accordingly, a minimum of 255 participants were required to conduct the study without design effect and non-response rate consideration. Then we add a 10.0% non-response rate to compensate for the non-responses of the participant and a design effect of 1.5 to compensate for the loss of efficient sample power, and we use the total population of the district as the reference population (N = 142,069). The total sample size was considered after checking various parameters of the measurements. Then, 421 participants were required to conduct the study.

### Sampling technique

The sample was selected using a two-stage sampling technique. First, among the 22 rural kebeles, six were selected randomly using the lottery method. Then, the sample was proportionally allocated among the selected kebeles based on the number of households and selected using a systematic random sampling technique based on the sampling frame. For each kebeles, using the kebeles administrators’ household list and the Community Health Information System (CHIS) as a sampling frame, a systematic selection of the allocated households was done. The interval was calculated by dividing the total households found in 6 rural kebeles by the total sample size (i.e., 8533 households divided by 421, which is 20). After obtaining the interval [20], we have selected the index household leveled 2 by the lottery method from the 20 households. Then, the study households in six rural kebeles were selected, beginning with the indexed household (leveled 2), by systematic random sampling technique in every 20th interval until the required sample was reached. Then, the data collectors went to the selected households to collect the required information from the heads of the households.

### Data collection tools and procedures

Pretested structured questionnaires were used to collect data from participants through face-to-face interviews. The tool contains five parts, such as 11 questions to assess socio-demographic and economic characteristics, 4 questions to assess knowledge of the participants, 19 questions to assess healthcare-related factors, 12 questions to assess income and wealth index, and 9 questions to assess willingness towards the community-based health insurance program, which had a scenario presentation with a benefit package of the scheme (Additional file 1). The tool was first prepared in English, translated to Hadiyyisa, and then returned to English to examine its consistency. Finally, the Hadiyyisa version of the questionnaire was used for data collection.

Data were collected from the heads of the selected households by six trained data collectors with BSc degrees, and two supervisors with master’s degrees were involved in the data collection process. Three days of training were given to data collectors and supervisors. The data collection process was supervised by the principal investigator and supervisor.

### Operational definition

#### Willingness to pay (WTP)

After giving consent, each participant received a comprehensive illustration of a standard CBHI scenario before being asked if they would be willing to pay for it. Then, the participant’s level of willingness to pay for the CBHI scheme was measured by asking whether the participant was willing to pay some amount of premium Ethiopian Birr (ETB) for the CBHI scheme during the data collection period [1], and the real amount of premium that they were willing to pay for the CBHI scheme was assessed using the double-bounded dichotomous choice Variant scenario in the contingent valuation method [17].

#### Knowledge of the CBHI

Based on the summative score of questions designed to assess knowledge of the benefit package and basic principles of the CBHI. Households that gave 70.0% or more correct responses were categorized as having good knowledge. And those who scored less than 70.0% on the knowledge questions were categorized as having poor knowledge [18, 19].

#### Wealth index

It was measured using a standard tool that had twelve questions. We observed high internal consistency among items (Cronbach’s α = 0.79), and we used principal component analysis using varimax rotation to determine composite wealth indexes and the wealth status of the participants. Using ownership of those 12 household items, principal component analysis was used to derive the household wealth index, which was classified into three categories: the poorest, middle, and richest tertiles [17].

#### Iddir participation

is an indigenous community insurance that existing to help victims deal with the financial burden of catastrophic events such as burial and sickness expenses [3]. Participation in Iddir has an impact on one’s willingness to pay for the CBHI scheme since it resembles having insurance because it incorporates formal health insurance’s risk-sharing and resource-pooling elements. When a participant officially joined the association, that was when they were deemed to have engaged in iddir [19].

### Data quality management

To maintain the quality of the data, a validated tool was used that was adapted from different studies conducted in developing countries, including Ethiopia, and we modified the tool to fit the context of our study. On the other hand, six data collectors and two supervisors received training regarding the goals and procedures of the data collection. Pre-testing was done to check the reliability of items in the questionnaires. After data collection, each questionnaire was given a unique code by the principal investigator. Any mistakes discovered after data entry were fixed once the original data were revised using the code number.

### Statistical analysis

The collected data were entered using Epi-Data version 3.1 data manager and it was checked for completeness, errors, missing values, and then it was exported into SPSS version 24 for analysis.

Principal Component Analysis (PCA) with varimax rotation was used to compute the wealth index of participants. These standardized scores were then used to create the break points that define the wealth index as low, medium, and high. Bivariable logistic regression analysis was computed to identify statistical association between each independent and dependent variable. The 95% confidence intervals were computed to assess the significance of bivariable associations. Variables with *P*-value < 0.25 in bivariable analysis were considered in multivariable analysis. Multivariable logistic regression analysis was conducted to identify factors associated with willingness to pay for CBHI scheme. Adjusted odds ratio (AOR) with 95% CI was used to report association and significance was declared at *P* < 0.05. The model fitness was checked by Hosmer and Lemeshow goodness of fit test.

### Ethical consideration

This study was reviewed and approved by the Institutional Review Board at the College of Medicine and Health Sciences at Hawassa University (Ref.no: IHRERC/018/2020). A permission letter was obtained from the SNNPR Health Bureau, Hadiya Zone Health Department, and Gombora District Health Office. Kebele leaders were also communicated with in advance. The study participants were informed clearly and in detail about the study, and a written consent was obtained. Their identities were hidden to assure confidentiality. To ensure this, all data collectors were instructed to maintain the confidentiality of every piece of data they collected.

## Result

### Socio-demographic characteristics of participants

A total of 407 household heads with 96.7% response rate were studied. Four hundred and four (99.3%) of the respondents were Hadiya in ethnicity. From the total participants, 145 (35.6%) were within the age range of 30 to 39 years. Most (77.9%) of the respondents were males. The majority (96.3%) of the respondents were married. One hundred nineteen (29.2%) of the participants had completed primary education while 81 (19.9%) were unable to read and write. The Protestant religion was practiced by 336 (82.6%), and 196 (48.2%) of the study participants’ wealth index was middle. With regard to occupation of the study participants, 278 (68.3%) were farmers. Nearly two-third (63.9%) of the studied households had a family size of less than or equal to five (Table 1).

**Table 1:** Socio-demographic and economic characteristics of the study participants, Gombora District, South Ethiopia, 2020

Variable	Category	Number	Percent
Sex of the household head	Male	317	77.9
	Female	90	22.1
Age of the respondents	≤29 years	69	17.0
	30–39 years	145	35.6
	40–49 years	126	31.0
	50–59 years	55	13.5
	60 years and above	12	2.9
Region of the respondent	Orthodox	64	15.7
	Protestant	336	82.6
	Catholic	7	1.7
Ethnicity of the respondent	Hadiya	404	99.3
	Gurage	3	0.7
Marital status of the respondent	Single	15	3.7
	Married	392	96.3
Occupational status	Farmer	278	68.3
	Housewife	55	13.5
	Merchant	74	18.2
Educational status	Unable to read and write	81	19.9
	Read and write	98	24.1
	Primary education	119	29.2
	Secondary education	109	26.8
Family size	Less than or equal to 5 More than 5	260 147	63.9 36.1
Wealth index	Poor	142	34.9
	Middle	196	48.2
	Rich	69	17.0

### Household awareness about community-based health insurance

It was assessed by 10 yes/no items measuring knowledge about the benefit packages and basic principles of the CBHI scheme. Based on that, three hundred sixty-nine (90.7%) of the respondents had heard about community-based health insurance (CBHI) with their source information being kebele leaders (36.4%), health extension workers (31.8%), health facilities (26.8%), radio (3.0%), neighbors (1.4%) and television (0.5%). Out of these 261 (64.1%) of the participants describe community health insurance programs, 340 (83.5%) explain the advantage of community-based health insurance and 333 (81.8%) of the respondents know the health care services under the community-based health insurance program.

### Health and health related characteristics of the respondents

Concerning the health status of the members of the studied households, 171 (42.0%) of the household members had chronic illness or disability while 179 (44.0%) of the households encountered some illness in the past 12months. The median medical expense was 500 ETB per year per household, with the maximum medical expense of 3000 ETB and minimum of 0 (zero) ETB. Majority (98.9%) of the households covered the health care cost by themselves and the rest 2 (1.1%) of households covered the health care cost by community. Finding of money to pay for medical expense was difficult for 105 (58.7%) of households who encountered illness in the family.

Seventy-three (40.8%) of the respondents were satisfied with the health care service and costs, and 78 (43.6%) of the households perceived the quality of health care as high. Regarding the distance between the respondents’ place of residence to the nearby health facility, the median time it took to reach the nearby health facility on foot was 30 min, with a range between 5 and 120 min.

Three hundred and ninety-six (97.3%) of the households participate in iddirs. Out of them, 271(68.4%) households were participating in one iddirs. The median contribution of the households to iddirs was 20 ETB per month, with range of 5–80 (Table 2).

**Table 2:** Health and health related factors of the study participants, Gombora District, South Ethiopia, 2020

Description		Frequency	Percent
Self-reported health status of the household	Poor	52	12.8
	Medium	172	42.3
	Good	183	44.9
Persons with chronic illness and/or disability in the household	Yes	171	42
	No	236	58
Any illness encountered during the past 12 months	Yes	179	44
	No	228	56
Bought medical treatment for recent episode	Yes	178	99.4
	No	1	0.6
Got treatment	Yes	167	93.3
	No	12	6.7
Place of treatment	Private Health Facility	18	10.8
	Public Health Center	48	28.7
	Public Hospital	79	47.3
	Traditional healer & local drug vendor	22	13.2
Reason for visiting health facility	The HF was physically accessible The HF was not expensive	62 21	37.3 12.7
	The health service was effective	77	46.4
	HF not too crowded	6	3.6
Reason for not getting treatment	No enough money	8	66.7
	Self-limiting	3	25
	Too far location	1	8.3
Coverage of the health care cost	Self	177	98.9
	Community	2	1.1
Satisfaction with health care service and costs	Very dissatisfied	15	8.4
	Dissatisfied	61	34.1
	Neutral	6	3.3
	Satisfied	73	40.8
	Very satisfied	24	13.4
Perceived quality of the health care service in the district	Very low	12	6.7
	Low	71	39.6
	Neutral	3	1.7
	High	78	43.6
	Very high	15	8.4
Concern of the household for covering health care costs	Very difficult	21	11.7
	Difficult	105	58.7
	Not difficult	53	29.6
Means of getting money for health care payment	Borrow from someone	37	20.7
	Assisted by relatives	5	2.8
	Under taken extra work	28	15.6
Means of getting money for health care payment	Sell capital assets (cows) Drew from the saving	82 27	45.8 15.1
Borrow money for medical costs within last year	Yes	85	47.5
	No	94	52.5
The nearest conventional health institution to the respondents’ home	Health center	289	71
	Private clinic	37	9.1
	Government hospital	81	19.9

### Willingness to pay

Among 407 households, 273 (67.1%) were willing to pay for community-based health insurance in the district while 134 (32.9%) respondents were not willing to pay. As illustrated in the Table 3 below, 47.6% of the study participants were willing to pay the first bid. In the follow up questions, 36 (13.2%) of the respondents who were willing to pay the first bid were also willing to pay the second higher bid and 166 (60.8%) of the respondents who were not willing to pay the first bid were not willing to pay the second lower bid. Among those willing to pay, the mean amount of money household heads was willing to pay was 178 (± 57) Ethiopian Birr (ETB) per house hold per year and the median amount was 200 Ethiopian Birr (ETB). Also, the minimum and maximum amount of money household heads willing to pay was 40 and 450 Ethiopian Birr (ETB) respectively. Most of the households, 205 (50.4%) prefer to pay the premium of community-based health insurance by annual flat rate payment in 2020. N.B. (1 USD = 27.74 ETB) (Table 3).

**Table 3:** Summary Statistics of double bounded dichotomous choice contingent valuation questions of study participants, Gombora District, 2020

Scenarios		Number	Percent
Accepting initial bid	Yes	130	47.6
	No	143	52.4
Accepting second higher bid	Yes	36	13.2
	No	237	86.8
Accepting second lower bid	Yes	107	39.2
	No	166	60.8
WTP some amount of premium	Yes	273	67.1
	No	134	32.9

### Factor affecting the willingness to pay for CBHI

Table 4 below shows the multiple logistic regression results on the relationship between respondent demographic, socioeconomic, health and health related factors and willingness to pay for the community-based health insurance scheme.

Wealth index, health status, educational status, family size, iddir participation, occupational status, and chronic illness had *p* ≤ 0.25 on bivariable analysis and these variables were subjected to multivariable logistic regression. In multivariable logistic regression analysis, wealth index, educational status, family size, iddir participation and chronic illness in the family of the respondents showed a significant association with willing to pay for the CBHI scheme.

Household heads in the highest wealth index had 2.78 times higher odds of willingness to pay (WTP) for community-based health insurance (CBHI) than those with lowest wealth index (AOR: 2.78, 95%CI: 1.54, 5.03). Household heads who can read and write had 3.2 times higher odds of WTP for CBHI than respondents with no formal education (AOR: 2.90, 95% CI: 1.39, 6.05).

Heads of households of more than five family members had 1.7 times more odds of WTP for the community-based health insurance than those with less than or equal to five family members (AOR: 1.76, 95% CI: 1.06, 2.92). Besides, heads of households who participates in less than two iddirs had 2.7 times more odds of WTP for community-based health insurance than those who participate in greater or equal to two iddirs participation (AOR: 2.66, 95% CI: 1.60, 4.42). Furthermore, heads of households in which at least one family member had a chronic illness had 1.94 times higher odds of WTP for community-based health insurance than family member of the households with no chronic illness (AOR: 1.94, 95% CI: 1.21, 3.12). (Table 4)

**Table 4:** Determinants of willingness to pay for the community-based health insurance, Gombora District, southern Ethiopia, 2020

Variables	Willingness to pay		COR (95% CI)	AOR (95% CI)
	Yes	No		
**Wealth index**				
Poor	107 (79.3%)	28 (20.7%)	1	1
Middle	97 (72.4%)	37 (27.6%)	1.19 (0.69, 2.06)	1.14 (0.61, 2.13)
Rich	69 (50.0%)	69 (50.0%)	2.99 (1.78, 5.03)	2.78 (1.54, 5.03) *
**Health status**				
Poor	26 (50.0%)	26 (50.0%)	2.81 (1.49, 5.31)	1.92 (0.94, 3.91)
Medium	112 (65.1%)	60 (34.9%)	1.51 (0.96, 2.37)	1.15 (0.69, 1.92)
Good	135 (73.8%)	48 (26.2%)	1	1
**Education**				
No formal education	60 (74.1%	21 (25.9%)	1	1
Read and write only	53 (54.1%)	45 (45.9%)	2.43 (1.28, 4.58)	2.90 (1.39, 6.05) *
Primary education	80 (67.2%)	39 (32.8%)	1.39 (0.74, 2.61)	1.48 (0.73, 2.98)
Secondary and above	80 (73.4%)	29 (26.6%)	1.04 (0.54, 1.99)	0.91 (0.44, 1.89)
**Family size**				
≤ 5 members	163 (62.7%)	97 (37.3%)	1	1
> 5 members	110 (74.8%)	37 (25.2%)	1.77 (1.13, 2.77)	1.76 (1.06, 2.92) *
**Iddir participation**				
< 2 iddir	101 (80.8%)	24 (19.2%)	2.66 (1.60, 4.42)	2.83 (1.61, 4.96) *
≥ 2 iddir	166 (61.3%)	105 (38.7%)	1	1
**Occupation**				
Farmer	186 (66.9%)	92 (33.1%)	1	1
Housewife	34 (61.8%)	21 (38.2%)	1.25 (0.69, 2.27)	1.04 (0.51, 2.13)
Merchant	53 (71.6%)	21 (28.4%)	0.80 (0.46, 1.41)	1.27 (0.48, 3.34)
**Chronic illness**				
Household with chronic illness	171 (72.5%)	65 (27.5%)	1.78 (1.17, 2.70)	1.94 (1.21, 3.12) *
Household without Chronic illness	102 (59.6%)	69 (40.4%)	1	1

NB: 1-Reference category, *-statistically significant at *p*-value ≤ 0.05, COR-Crude Odds Ratio, AOR-Adjusted Odds Ratio

## Discussion

A community-based health insurance scheme was created to provide financial protection against the rising costs of health-care utilization, primarily among lower socioeconomic groups. However, studies have found that just a small percentage of the population is taking advantage of the program, particularly in developing countries like Ethiopia. This study was proposed to show the magnitude and determinants of willingness to pay for community-based health insurance in Gombora District, southern Ethiopia. Among the participants nearly 67.0% responded that they would pay for the community-based health insurance scheme. Which is nearly comparable with the study done in Fogera District, North West Ethiopia and Kewiot and Eftratana Gedem district of Amhara Region, Ethiopia [16, 20]. The mean amount of money willing to pay per household per year was 178.41 ETB per person per year. Factors such as wealth index, educational status, family size, indigenous community insurance (iddir) participation and presence of chronic illness in the household are significantly associated with willingness to pay for community-based health insurance.

The mean amount money willing to pay per household per year in this study was 178.41 ETB per person per year. It accounted about 40.0% of Ethiopian national health spending per capita ($16.1 USD) [21]. Also the mean amount money willing to pay per household per year in this study is greater than study in North Central Nigeria [17]. However, this is lower than study done in Fogera District, North West Ethiopia [20]. The mean amount money willing to pay for community-based health insurance in North Central Nigeria and Fogera District was 522.0 ± 266.3-naira (3.48 ± 1.78 USD) and 187 Ethiopian Birr (ETB) (1.95 USD) respectively. Also the Study conducted in Wuhan, China by Till Barnghausen et al. found that the mean WTP among informal sector workers was a correspondent of 4 USD per person per month for community health insurance (48 USD per person per year) [16]. This difference compared to our finding might be due to the differences in existing socio-economic situations, health insurance experience and level of industrialization which clearly affect the earning power.

Households with rich wealth index had around three times higher odds of willingness to pay for community-based health insurance as compared to households with poor wealth index. This is in line with a study done on willingness to pay for community-based health insurance among households in the rural community of Fogera District, North West Ethiopia [20], and study conducted on a systematic review of factors that affect uptake of community-based health insurance in low-income and middle-income countries by Adebayo et al. [21]. This result also agrees with the study conducted by Johannes Paul in rural developing countries as an example of Senegal on the impact of health insurance on the access to health care and financial protection [22]. This might be as the result from their ability to pay the minimum amount of community-based health insurance scheme.

The results of the multiple logistic regression analysis revealed that respondents with larger families were 1.7 times more likely willing to pay community-based health insurance than respondents with smaller households. This result agrees with the study done on willingness to pay for community-based health insurance among households in the rural community of Fogera District, North West Ethiopia [20], in Kewiot and Eftratana Gedem districts of Amhara Region, Ethiopia [16], and Nigeria (Edo state) by Oriakhi et al. [23], where the willingness to pay of the rural community was influenced by household size. This might be as a result of the high financial burden faced by large household when seeking health care services.

Educational level of the household was found to be another factor to increase the willingness to pay for community-based health insurance. The result was supported with the study conducted on willingness to pay for community-based health insurance among households in the rural community of Fogera District, North West Ethiopia [20], Willingness to pay for community health insurance and its determinants among household heads in rural communities in North Central Nigeria [17]. Educated household heads are more aware of the benefits of making regularly insurance payments to reduce the possibility of catastrophic medical expenses during illness [24]. This might be households who had education would care more about their health and they can understand about the reform so that education might support in making informed decision about the scheme.

Another attractive variable in this study is whether a household has chronic disease or not. Heads of households in which at least one family member had a chronic illness had about two times higher odds of WTP for community-based health insurance than family member of the households with no chronic illness. The results show as the family does have a chronic illness so that they have more willing to pay community-based health insurance. The result was in line with the study done on the feasibility of health insurance schemes for community-based groups by Isreal Fekade in Addis Ababa City, Ethiopia [14]. This might be due to the reality that these households have a greater awareness of the extent of the loss at whatever time a health care service is needed by a member with a chronic illness.

Households who participate in one iddir (indigenous community insurance) were three times more likely willing to pay community-based health insurance than those who participates in greater or equal to two iddir. The result was supported by the study done in Jimma town, western part of Ethiopia as iddir participation increases willingness to pay for community health insurance. However, they did not study the reason why the households participate in less than two iddir were willing to pay more [24]. This might be households who participate in one iddir are more vulnerable to risks, so that they prefer to pay more to protect themselves in the case of voluntary health insurance. It might also be attributable to the reality that participation in iddirs may develop bond and team spirit among the members which results in the spirit of helping each other than those who are not.

### Implication of the study

This study has some important implications. The proportion of households willing to pay for the scheme was low as compared to the goal of universal health coverage. The willingness to pay for CBHI was influenced by the wealth index, educational status, family size, iddir (indigenous community insurance) participation, and chronic illness of the households. Therefore, the government had better consider household family size and wealth status when setting the premium load. Moreover, the government should create awareness in the community about the scheme to scale up the program.

### Strength and limitation of the study

As the strength of the study, it used the double-bound dichotomous choice variant of the contingent valuation method, which helps to reduce response bias. Since it is statistically more efficient than the single-bounded format and the confidence interval of the estimated willingness-to-pay is narrower, However, this study has its own limitations since we used a cross-sectional study, which is weak to explore determinants of willingness towards community-based health insurance programs.

## Conclusion

About two-thirds (67.1%) of the households were willing to pay for a community-based health insurance scheme. Household’s willingness to pay in a community-based health insurance scheme was found to be significantly influenced by poor household wealth status, household heads who cannot read and write, households with less than or equal to five family members, households who participate in greater or equal to two indigenous community insurance participations, and the absence of chronic illness within the household. Therefore, factors affecting the household’s willingness to pay should be emphasized to enhance community health insurance enrollment, which leads to universal health coverage. In addition, massive community mobilization needs to be done to strengthen and increase the membership of households during the implementation of the CBHI scheme by giving priorities to individuals with the characteristics of the mentioned factors. Therefore, while computing the amount of premium, it is critical to account for variations in WTP for CBHI schemes that appear across different groups.

### Electronic supplementary material

Below is the link to the electronic supplementary material.

**Additional file 1:** Questionnaire for research on Determinants of willingness to pay for community-based health insurance scheme among households in rural community of southern Ethiopia, 2020

## Data Availability

The datasets used and/or analysed during the current study are available from the corresponding author on reasonable request.

## Abbreviations

CBHF: Community Based Health Financing
CBHI: Community Based Health Insurance
CHI: Community Health Insurance
CI: Confidence interval
CVM: Contingent Valuation Method
DBDCV: Double Bounded Dichotomous Choice Variant
EHSFR: Ethiopia Health Sector Financing Reform
ETB: Ethiopian Birr
FMOH: Federal Ministry of Health
HH: Household
HSFRP: Health Sector Financing Reform Program
LMIC: Low and Middle Income Country
OOP: Out-Of-Pocket Payment
SNNP: Southern Nations, Nationalities and People’s
SPSS: Statistical Software for Social Science
USD: United States Dollar
WTP: Willingness to Pay
WTJ: Willingness to Join
