# Supplementary material for: Determinants of willingness to pay for community-based health insurance scheme among households in rural community of southern Ethiopia
Source: BMC Health Serv Res. 2023 Dec 6;23:1365. doi: 10.1186/s12913-023-10406-w (PMC10698878; doi:10.1186/s12913-023-10406-w)
Supplement: Supplementary file 1 — Additional file 1: Questionnaire for research on Determinants of willingness to pay for community-based health insurance scheme among households in rural community of southern Ethiopia, 2020 [file 12913_2023_10406_MOESM1_ESM.docx]

**QUESTIONNAIRE USED IN THE STUDY**

**Questionnaire for research on Determinants of willingness to pay for community-based health insurance scheme among households in rural community of southern Ethiopia, 2020**

**Introduction**

Hello, my name is ______________; I am one of the data collectors in this study. We are interviewing household heads here in ______________kebeles in order to find out information about willingness to pay for the newly proposed community-based health insurance. To attain this purpose, your honest and genuine participation by responding to the questions prepared is very important and highly appreciated.

**Confidentiality and consent**

We would like you to answer some personal questions. Your answers are completely confidential and participation is voluntary. Your name will not be written on this form, and will never be used in connection with any of the information you tell me. You don’t have to answer any question if you do not want to and you can stop the interview at any time.

However, your honest answer to these questions will help us to better understand the situation and will contribute to improve the health status of the community by identifying the demand and ability to pay for community-based health insurance and determinant factors then presenting to the policy makers so as to adjust the schemes with the local context. We would greatly appreciate your help in participating in this study. The interview will take 25 to 30 minutes to ask the questions.

**Would you be willing to participate?
□ Yes, proceed****□ No, good bye. Thank you for your cooperation!**

**CONSENT FORM:**

I have been explained all information and procedures that are part of this research study and I
have understood the same. I understand that the research imposes no risk on my life and
therefore no compensation would be provided. I hereby agree to participate in this research study and give my voluntary consent. I hereby also give rights to the researcher for collecting the data that are required for the study.

_____________________________________________________________________

(Signature of interviewer certifying that informed consent has been given by respondent).

Date of data collection_________________________________

Name of data collector__________________________ Signature_________________

Name of supervisor_____________________________ Signature_________________

**Questionnaire for research on Determinants of willingness to pay for community-based health insurance scheme among households in rural community of southern Ethiopia, 2020**

Questionnaire No _________________________________

Interviewer’s Name________________________________
Supervisor’s Name_________________________________
Date of interview: DD_______MM______ YYYY________
Interview times taken: started __________ended__________
Address: Woreda ___________________________________

| **Part 1- Household (Socio-demographic and socio-economic) questionnaire** | | | | | | | |
| --- | --- | --- | --- | --- | --- | --- | --- |
| Kebeles --------------  House no------------- | | | | | | | |
| Q | | Questions | RESPONSE CODE (CHECK THE APPROPRIATE OPTION OR ENTER NUMBERS) | | | | Skip |
| Q101 | | What is your age? | (in years) ____________ | | | |  |
| Q102 | | What is the sex of the respondents? | 1. Male  2. Female | | | |  |
| Q103 | | What is your Religion? | 1. Orthodox  2. Muslim 3. Protestant  4. Catholic 5. Other (specify) __________ | | | |  |
| Q104 | | What is your Ethnicity? | 1. Hadiya  2. Amhara 3. Guragie  4. Kambata  5. Siltie 6.Other (specify)__________ | | | |  |
| Q105 | | What is your marital status? | 1. Single  2. Married  3. Widowed  4. Divorced | | | |  |
| Q106 | | What is your occupation status? | 1. Farmer  2. Housewife 3. Merchant  4. Laborer 5. Student  6. Other (specify)__________ | | | |  |
| Q107 | | What is your educational status? | 1. Unable to read and write  2. Read and write 3. Primary education  4. Secondary education  5. Tertiary school | | | |  |
| Q108 | | What is the size of the family under the household? | Number of family size _________  Male __________  Female __________ | | | |  |
| Q109 | | Do you participate in iddirs? | 1. Yes  2. No | | | | If “no”, skip to Q 201 |
| Q110 | | If yes, for Q109, How many iddirs do you participate in? | Number ______________ | | | |  |
| Q111 | | How much do you contribute for all iddirs you participate in per month? | Amount in birr _________ | | | |  |
| **Part-2 awareness and knowledge questions** | | | | | | | |
| Q201 | | Have you ever heard about community-based health insurance? | 1. Yes  2. No | | | | If “no”, skip to Q 203 |
| Q202 | | From whom you heard? | 1. Health extension workers 2. Radio  3. Television 4. kebele leader  5. From my neighbour 6. Health facility  7. Other (specify) ___________ | | | |  |
| Q203 | | What are the benefits of community-based health insurance? | 1. Risk sharing among members 2. Assuring equity 3. Solidarity  4. Community participation  5. Don’t know  6. Other (specify) __________ | | | |  |
| Q204 | | What kinds of services do you think you will get if you are a member of the community-based health insurance? | 1. Outpatient service 2. Inpatient service 3. Laboratory and imaging service 4. Genetic drug service 5. Surgery service 6. Referral service from health centers to Hospitals  7. Others (specify) ___________ | | | |  |
| **Part 3- Health and health related questions** | | | | | | | |
| Q301 | | How do you rate the health status of yours, spouse and children age of less than 18 years in your household? | | | 1. Very poor  2. Poor 3. Medium  4. Good 5. Very good | |  |
| Q302 | | Do you or other member of the household have chronic illness and/or disabled? | | | 1. Yes  2. No | |  |
| Q303 | | Have any member of the family encountered any illness during the last 12 months? | | | 1. Yes  2. No | | If “no”, skip to Q 305 |
| Q304 | | How many of the members were ill? | | | The number of the ill __________ | |  |
| Q305 | | Did you seek medical treatment for the last 12 month episode? | | | 1. Yes  2. No | | If “no”, skip to Q 318 |
| Q306 | | Did you get treatment? | | | 1. Yes  2. No | | If “no”, skip to Q 309 |
| Q307 | | Where did you get treatment? | | | 1. Self-treatment 2. Local drug vender 3. Private Heath Facility 4. Public health center 5. Public hospital  6. Traditional healer  7. Other (specify) __________ | |  |
| Q308 | | Why did you go there? | | | 1. The HF was physically accessible 2. The HF was not expensive 3. The health facility not too crowded 4. The health service was courteous 5. The health service was efficacious/effective  6. Other (specify) __________ | |  |
| Q309 | | Why did not you get treatment? | | | 1. Considering the illness is self-limiting 2. No enough money  3. Didn’t know anywhere to go 4. Didn’t have time to go to health facility 5. Too far to go health facility 6. Other (specify) __________ | |  |
| Q310 | | How much was total health care cost of the household for the treatment in the last 12 months? | | | Amount in Birr __________ | | If ‘not paid’ skip to Q312 |
| Q311 | | Who covered the health care cost? | | | 1. Self  2. Government/free 3. Community 4.Other (specify) ________ | |  |
| Q312 | | How was your satisfaction with health care service and costs? | | | 1. Very dissatisfied  2. Dissatisfied 3. Neutral  4. Satisfied 5. Very satisfied | |  |
| Q313 | | How did you perceive quality of the health care service in this area? | | | 1. Very low  2. Low 3. Neutral  4. High  5. Very high | |  |
| Q314 | | How did you see finding money to pay for the health care? | | | 1. Very difficult  2. Difficult 3. Not difficult | |  |
| Q315 | | If paying for a medical expense was difficult, how did you get it? | | | 1. Drew from the savings 2. Borrow from someone 3. Assisted by relatives 4. Undertaken extra work 5. Sell capital assets such as livestock 6. Cut back on other things, food, drink, cloth etc.  7. Others (specify) __________ | |  |
| Q316 | | Did you borrow any money from relatives or other people to cover medical costs within the last year? | | | 1. Yes  2. No | | If “no”, skip to Q 318 |
| Q317 | | How much did you borrow? | | | Amount borrowed in Birr __________ | |  |
| Q318 | | What is the nearest health facility to your home that is usually used for medical care? | | | 1. Health center  2. Clinic (Private) 3. Hospital (Gov.)  4. Others specify __________ | |  |
| Q319 | | How long does it take to reach the nearby HF from your home? | | | Time in minutes ___________ | |  |
| **Part 4-Income and wealth index questions** | | | | | | | |
| Q401 | Approximately, how much of these products did your household produced and sold during the last 1 year? | | | | 1. Banana in Birr 2. Chat sold in Birr 3. Wheat (in quintals) 4. Potato (in quintals) 5. Butter (in Birr) 6. Others (specify) ____________ | |  |
| Q402 | How many of these animals do your household own? | | | | 1. Milk cows, oxen or bulls? 2. Goats?  3. Sheep? 4. Chickens? 5. Beehives? 6. Other (specify) _____________ | |  |
| Q403 | Does your household have? | | | |  | |  |
|  | Q403.1- Functional radio/tape? | | | | 1. Yes  2. No | |  |
|  | Q403.2- Horse/mule /Donkey? | | | | 1. Yes  2. No | |  |
|  | Q403.3- Cotton/sponge/spring mattress? | | | | 1. Yes  2. No | |  |
|  | Q403.4- Bed | | | | 1. Yes  2. No | |  |
|  | Q403.5- Mobile phone | | | | 1. Yes  2. No | |  |
| Q404 | What kind of latrine does your family have? | | | | 1. None  2. VIP 3. Pit latrine with slab 4. Pit latrine without slab 5. Other (specify) ___________ | |  |
| Q405 | What is the type of roof of the house? | | | | 1. Corrugated sheet 2. Thatch roof 3. Other (specify) ____________ | |  |
| Q406 | How many rooms does your household have for sleeping only? | | | | Number of rooms’ ___________ | |  |
| Q407 | Do you have a separate kitchen room? | | | | 1. Yes  2. No | |  |
| Q408 | Do you have separate rooms for cattle? | | | | 1. Yes  2. No | |  |
| Q409 | What is the wall of your residence house made of? | | | | 1. Wooden structure  2. Mud 3. Other (specify) __________ | |  |
| Q410 | What is the floor of your residence house made of? | | | | 1. Mud  2. Cemented  3. Hard wood  4. Ceramic tile  5. Others (specify) ____________ | |  |
| Q411 | What is the total farm size owned by the household in hectares? | | | | Size in hectares ____________ | |  |
| Q412 | What is the source of water for drinking? | | | | 1.Un-protected spring 2.Protected spring  3.River water 4.Private pipe water  5.Public tap/standpipe  6.other (specify) ______________ | |  |
| **Part-5: Community-based health insurance scenario** | | | | | | | |
| I**ntroduction**  Sickness needs to be treated immediately and it is not possible to wait. If you do not have the money available, you will need to borrow it from your neighbour or sell your sheep or chickens. While you run around trying to get the money together, the sick person suffers. And many times, it happens that you come back with the money only to find out that the sick person has died.  In order to solve this financial problem, The Federal Democratic Republic of Ethiopia, Ministry of Health is planning to set community- based health insurance in your community, if the community members want to be included in such programs. If you join the insurance and pay an annual premium, you do not need to pay for the following health service provided in your district area for a period of one year for your family members (the head of the house hold, the spouse and children age of less than 18 years).  **Benefits package**  **Drugs:** all essential and generic which you already buy in your pharmacy, either at the health center level or at district Hospital level. The insurance may pay for drugs sold in accredited private drug stores.  **Laboratory tests:** all costs of laboratory tests that have been prescribed by the public health agent are covered if they are being carried out in accredited public or private health facilities.  **Inpatient stays:** when you are hospitalized, the insurance will cover the expenses. Urgent transport by ambulance from your village to the primary Hospital in the locality will also be covered.  **Surgery:** general surgery and delivery complications, the extraction of teeth and circumcision are included. But surgeries for cosmetics purposes such as plastic surgery are not included. X-ray will be covered if the Doctor thinks it is necessary.  Organization of insurance scheme committee selected from your kebeles workers and the kebeles chairperson will manage the scheme. The premiums will be kept in a bank. The committee chair and a treasurer have a right to withdraw the money from the bank and to pay heath facilities. An annual audit will ensure that funds are used rationally. The committee will give a financial report of the scheme to local government every year.  **Enrolment and payment**  You need to pay an annual premium for joining the insurance and 10.00 ETB for registration. Credit is not allowed. After you pay the premiums, then you can enjoy the benefits of insurance after 3 months. If you do not pay the premiums, you have to pay for all service ‘out-of-pocket’. For example, if you suffer from typhoid, you have to pay for the treatment and laboratory diagnosis services.  **Reimbursement procedure**  Insured patient do not need pay to see a Doctor for the services that are covered by the insurance. The money will be paid by the committee. But patients will have to pay for the services not covered by the insurance. | | | | | | | |
| **Part-5: Willingness to pay for community-based health insurance** | | | | | | | |
| Q501 | | Based on the above scenario, will you pay for the community-based health insurance established in your community? | | 1. Yes 2. No | | If “no” skip to Q.506 | |
| Q502 | | Which initial bid have you taken? | | 1. 75 ETB  2. 100 ETB 3. 180 ETB  4. 250 ETB 5. 300ETB  6. 400 ETB  7. 500 ETB | |  | |
| Q503 | | Will you pay the initial bid as annual premium per household for community-based health insurance? | | 1. Yes 2. No | | If “no”, skip to Q 504 | |
| Q504 | | Will you pay if the premium is double of the specified amount of Birr per year per household? | | 1. Yes 2. No | | If “no”, skip to Q 507. | |
| Q505 | | Maximum how much shall you pay per year per household as a premium for community-based health insurance? | | Specify the amount in Birr __________ | |  | |
| Q506 | | Will you pay if the premium is half of the specified Birr amount per year per household? | | 1. Yes 2. No | | If “yes”, skip to Q 509 | |
| Q507 | | Maximum, how much shall you pay per year per household? | | Specify the amount in birr _________ | | Go to Q 508, if answer = 0 birr; Go to 509 if>0 birr. | |
| Q508 | | Why is your household not willing to pay for the scheme? | | 1. Doubt the management of the fund 2. It is the responsibility of the government to pay for such a program 3. Because of lack of money 4. Other members of the society should pay for the programs 5.the quality of health service is poor 6. Out of –pocket payment is better than CBHI scheme 7. Other (specify) _____________ | | Stop the interview here | |
| Q509 | | How frequently do you want to pay the yearly premium? | | 1. Annual flat rate 2. Bi-annual flat-rate 3. Quarterly a year flat-rate 4. Monthly 5. Other (specify)_________ | |  | |
| **Thank you for your response!!!**  **The time interview ended ________________** | | | | | | | |
